# Current research on head and neck cancer-associated long noncoding RNAs

**DOI:** 10.18632/oncotarget.22608

**Published:** 2017-11-22

**Authors:** Wei Song, Yimin Sun, Jie Lin, Xiaoqin Bi

**Affiliations:** ^1^ State Key Laboratory of Oral Diseases, West China School of Stomatology, Sichuan University, Chengdu, Sichuan 610041, P.R. China; ^2^ Department of Dental Anesthesiology, West China Hospital of Stomatology, State Key Laboratory of Oral Diseases, Sichuan University, Chengdu, Sichuan 610041, P.R. China; ^3^ Department of Head and Neck Oncology, West China Hospital of Stomatology, State Key Laboratory of Oral Diseases, Sichuan University, Chengdu, Sichuan 610041, P.R. China

**Keywords:** head and neck cancer, long noncoding RNA, cancer progression, clinical implication

## Abstract

Head and neck cancers (HNC) are one of the ten leading cancers worldwide, including a range of malignant tumors arising from the upper neck. Due to the complex mechanisms of HNC and lack of effective biomarkers, the 5-year survival rate of HNC has been low and the mortality rate has been high in recent decades. Long noncoding RNAs (lncRNAs), noncoding RNAs longer than 200 bps, are a focus of current cancer research, closely related to tumor biology. LncRNAs have been revealed to be aberrantly expressed in various types of HNC, and the dysregulated lncRNAs participate in HNC progression and induce malignant behavior by modulating gene expression at diverse levels. This review will focus on the functions and molecular mechanisms of dysregulated lncRNAs in HNC tumorigenesis and progression, as well as their diagnostic, therapeutic or prognostic implications in HNC.

## INTRODUCTION

Head and neck cancers (HNC) are one of the ten leading cancers worldwide originating from the upper neck, including the oral cavity, tongue, hypopharynx, nasopharynx, larynx, and thyroid [1–4]. More than 90% of the cancer cases of squamous origin, forms the most common histological subtype of HNC, referred to as head and neck squamous cell carcinoma (HNSCC) [5]. Smoking, alcohol consumption are the most common incentives of HNSCC, and the human papillomavirus (HPV) infection also contributes to its occurrence [6–9].

Oral squamous cell carcinoma (OSCC) is the fourth most common cancer in the world, accounting for more than 90% of oral cancer [10, 11]. Due to the consumption of alcohol, tobacco and areca chewing, males’ incidence of OSCC is significantly higher than females’ [10]. Laryngeal squamous cell carcinoma (LSCC), a malignant cancer arising from the upper respiratory tract, consists of more than 95% of laryngeal cancers [12]. LSCC mostly occurs in glottic (60%) or supraglottic regions, and only 5% cases are subglottic [12]. Currently, the main methods for LSCC treatment include surgical intervention, radiotherapy and chemotherapy. Most of them may damage physiological functions like swallowing or speaking. Tongue squamous cell carcinoma (TSCC) is the major subtype of oral cancer with a high rate of proliferation and recurrence, and is famous for its aggressive, rapid local invasion and spread [13–15]. Hypopharyngeal squamous cell carcinoma (HSCC), a relatively rare type of HNC, arises from mucosal lesions of the upper aerodigestive tract [16]. HSCC has rather dismal prognosis and the lowest survival rate compared with other types of HNSCC due to poor locoregional control and lack of obvious early symptoms [17–20]. Nasopharyngeal carcinoma (NPC) is a prevalent malignant tumor and particularly occurs in specific geographic localities including North Africa, Southeast Asia, and Southern China [21, 22]. NPC can be divided into 2 types (differentiated and undifferentiated NPC), and the primary histologic type is the latter [23]. Resulted from its anatomical position (near the skull) and the unobvious symptoms, the early diagnosis of NPC is so difficult that patients are mostly diagnosed in advanced and/or metastatic stages [24, 25]. Thyroid cancer (TC) is the most common endocrine cancer, which can be classified into two histological types according to variants in tumor origins. Tumors derived from follicular thyroid cells dominate thyroid carcinomas, including papillary thyroid cancer (PTC), follicular thyroid cancer (FTC), and anaplastic thyroid cancer (ATC). Parafollicular C cell-derived tumors only accounts for a small part of all the cases, among which medullary thyroid cancer (MTC) takes up the majority. PTC and FTC form the type known as differentiated thyroid cancer (DTC), while MTC and ATC are collectively classified as poorly differentiated thyroid cancer (PDTC) [26, 27].

The initiation and progression of HNC is a rather complex multistep process involving genetic and epigenetic changes and dynamic alternations in the genome. Both the complex mechanisms of HNC and the lack of effective biomarkers contribute to dismal prognosis. Thus, there is a compelling need to elucidate the mechanisms of HNC and explore predictive biomarkers so that cancers can be diagnosed early and patients can be provided with efficient therapeutic strategies [28–30].

Previously, noncoding RNAs (ncRNAs) were widely regarded as the “junk” or “noise” of genetic transcription since ncRNAs lack protein-coding potential; but in recent years, with the development of DNA sequencing technologies, ncRNAs are reported to participate in regulating expression of coding genes during the whole gene expression process [31]. According to the size of the transcript, non-coding RNAs are categorized into two groups: short ncRNAs (< 200 nt) and long ncRNAs (lncRNAs) (> 200 nt). LncRNAs are generally located in the nucleus, with their expression patterns highly tissue-specific [32, 33]. LncRNAs are a focus of current cancer research, closely related to tumor biology. Experimental results have demonstrated that a growing number of lncRNAs are found dysregulated in HNC and some can be the driving force of malignant transformation via regulating HNC cell viability and motility [34, 35]. Aberrantly expressed lncRNAs contribute to multiple biological processes such as cellular growth, proliferation and differentiation, apoptosis, and tumorigenesis by modulating gene expression at epigenetic, transcriptional, post-transcriptional, translational and post-translational levels [35–37].

Aside from those well-known HNC-associated lncRNAs, the expression and functions of numerous lncRNAs in HNC are still elusive. Some lncRNAs have been found to play pivotal roles in HNC initiation and progression, suggesting they might function as novel biomarkers and therapeutic targets to provide more effective diagnosis, prognosis and treatment for HNC patients [35, 38]. Therefore, in the review we outline the extensively studied lncRNAs and several recently discovered HNC-related lncRNAs, and try to elaborate the role they play in HNC along with the mechanisms of HNC pathogenesis.

### The lncRNAs extensively associated with HNC

Previously, long noncoding RNAs in head and neck cancers have been extensively studied. The expression levels of lncRNAs in cancer cells are related to different stages of cancer and cancer progression. Here, we classify lncRNA according to different tumor types, including HNSCC, NPC, TC, TSCC, OSCC and LSCC, and the effects of tumor-associated lncRNAs and their corresponding downstream molecules on the tumor are described respectively.

### HNSCC-associated lncRNAs

#### HOTAIR

LncRNA HOX transcript antisense RNA (HOTAIR), with a full-length sequence of 2.2 kb, is transcribed from the antisense strand of HOXC gene cluster on chromosome 12. Mounting evidence has revealed that HOTAIR, as a key epigenetic regulator, plays a pivotal role in initiation and progression of human malignant tumors, like esophageal squamous cell carcinoma, colorectal cancer and pancreatic cancer [39–41]. Also, the expression level of HOTAIR has been found significantly increased in HNC tissues than in paired adjacent non-tumor tissues.

Research has suggested a correlation between the aberrant expression of HOTAIR and cell viability. HOTAIR depletion can lead to cancer cell apoptosis *in vitro*, and decelerate tumor growth via activating mitochondrial-related cell death pathway in HNSCC both *in vitro* and *in vivo* [42]. Previous research has verified that the network consisting of lncRNAs, RNA binding proteins (RBPs) and miRNAs has tremendous implications during the HNSCC progression. There is a feed-forward regulatory loop between HOTAIR and HuR [43], an RBP participating in the post-transcriptional regulation of target genes [44]. HOTAIR could increase HuR expression via acting as a competing endogenous RNA (ceRNA) of miR-7. Conversely, HuR could enhance the sponge activity of HOTAIR and up-regulate it through miRNA recruitment [43]. Both HOTAIR and HuR are over-expressed in HNSCC, associated with the promotion of cell viability, metastasis and invasion [43].

#### H19

LncRNA H19, transcribed from chromosome 11p15.5, is becoming a hotspot in cancer research and is mainly located in cytoplasm. The H19 gene is abundantly expressed in mesoderm- and endoderm-derived tissues at embryonic stage, while it is not expressed in most parts of the body except cardiac and skeletal muscle after birth and is re-expressed in the tumor tissues [45, 46]. H19 expression is directly induced by c-myc in various cancers [47, 48].

H19 and Insulin-like growth factor 2 (IGF2), the adjacent reciprocal imprinted genes in the same locus, are expressed from the maternal and paternal allele, respectively. Loss of imprinting (LOI) at the H19 and IGF2 locus has been manifested to constitute a novel oncogenic mechanism of HNC. IGF2 and H19 were imprinted in all the normal squamous epithelium of 49 HNSCC samples examined (77% of 64 samples), while 12 of 32 samples(37.5%) confirmed LOI at the H19 gene and 11 of 27 samples (40.7%) proved LOI at the IGF2 gene [49]. Furthermore, the IGF2/H19 imprinting changes were correlated with juvenile nasopharyngeal angiofibroma (JNA). IGF2 overexpression was observed in 8 out of 22 cases (36.4%) and 7 out of 19 cases (36.8%) showed H19 overexpression [50]. Accumulating evidence has shown that lncRNA H19 is up-regulated in various human malignant tumors. H19 and its mature product miR-675 are significantly over-expressed in HNSCC, leading to higher invasive capability, worse overall survival (OS) and higher risk of tumor recurrence [51].

#### HNGA1

Aerobic glycolysis, a common inducement of cancer malignancy, has been recently reported to play a significant role in tumorigenesis and tumor development in HNSCC. A novel lncRNA, HNSCC glycolysis-associated 1 (HNGA1), has been found associated with HNSCC glycolysis and can regulate cell proliferation in HNSCC [52]. The expression of HNGA1 is significantly up-regulated while miR-375 is down-regulated in tumor tissues. Both of them are related to glycolysis: overexpression of HNGA1 promoted glycolysis in tumor tissues, whereas the ectopic expression of miR-375 has the opposite effect [52]. Moreover, their expressions in tumor tissues show a certain correlation, suggesting that there might be a specific mechanism of their joint regulation in the occurrence and development of HNSCC [52]. Previous research has revealed the role of SCL2A1, one of the gene encoding a major glucose transporter in the plasma membrane, in the HNSCC progression. Highly expressed miR-375 can directly inhibit the expression of SCL2A1 by binding to its 3’-UTR region, which has an inhibitory effect on tumor cell glycolysis and thereby inhibits cell proliferation. HNGA1 might serve as an endogenous “sponge” by competing for miR-375 binding site to eliminate the inhibitory effect of miR-375 on SCL2A1 expression and promote cell proliferation in HNSCC cells [52]. Similar mechanism has been reported in their other studies, and lncRNA-p23154 can modulate the glycolysis and proliferation of OSCC via repressing miR-375 expression [53].

#### PTENP1

PTENP1, a conserved pseudogene of PTEN located on chromosome band 9p21, is highly homologous to its ancestral gene PTEN [54]. Accumulating evidence has revealed that the expression levels of PTENP1 and PTEN are positively correlated, and both are significantly down-regulated in HNSCC cell lines compared with adjacent non-tumor cells [55]. The decreased PTENP1 expression in multiple cancers is mainly resulted from two reasons: PTENP1 promoter methylation and copy number reduction of genomic PTENP1. In HNSCC cell lines, 4 of 5 cell lines (80%) has been observed copy number reduction (completely or partially) in the PTENP1 locus, suggesting copy number alterations play a pivotal role in PTENP1 expression [55]. Ectopically expressed PTENP1 decelerates cell growth, colony formation, metastasis and invasion, and thus attenuates HNSCC tumorigenesis [55].

#### PANCR

PANCR is a lncRNA adjacent to the PITX2 gene which was first found in cardiomyocytes regulating the expression of splice variant PITX2C [56]. PITX2 is a homeobox gene located on chromosome 4q25 and encodes four isoforms (PITX2A, PITX2B, PITX2C, PITX2D) that are involved in the development of the structure prior to transcription [57]. In prostate cancer, hypermethylation of PITX2 is associated with a significant risk of disease progression [58, 59]. In contrast, the overexpression of hypomethylation of PITX2 may lead to tumor progression in ovarian and thyroid cancers [60]. The samples showed PITX2 methylation, whereas PANCR was found highly methylated in HNSCC tissues [61]. In HNSCC, PITX2 hypermethylation leads to a reduction in incidence, whereas PANCR hypermethylation is related to a higher risk of death in patients [61]. Additionally, high PITX2 methylation was related to p16 expression and prolonged survival in patients with p16 positive and PITX2 hypermethylation in HNSCC [61].

#### Application

**Figure 1:**
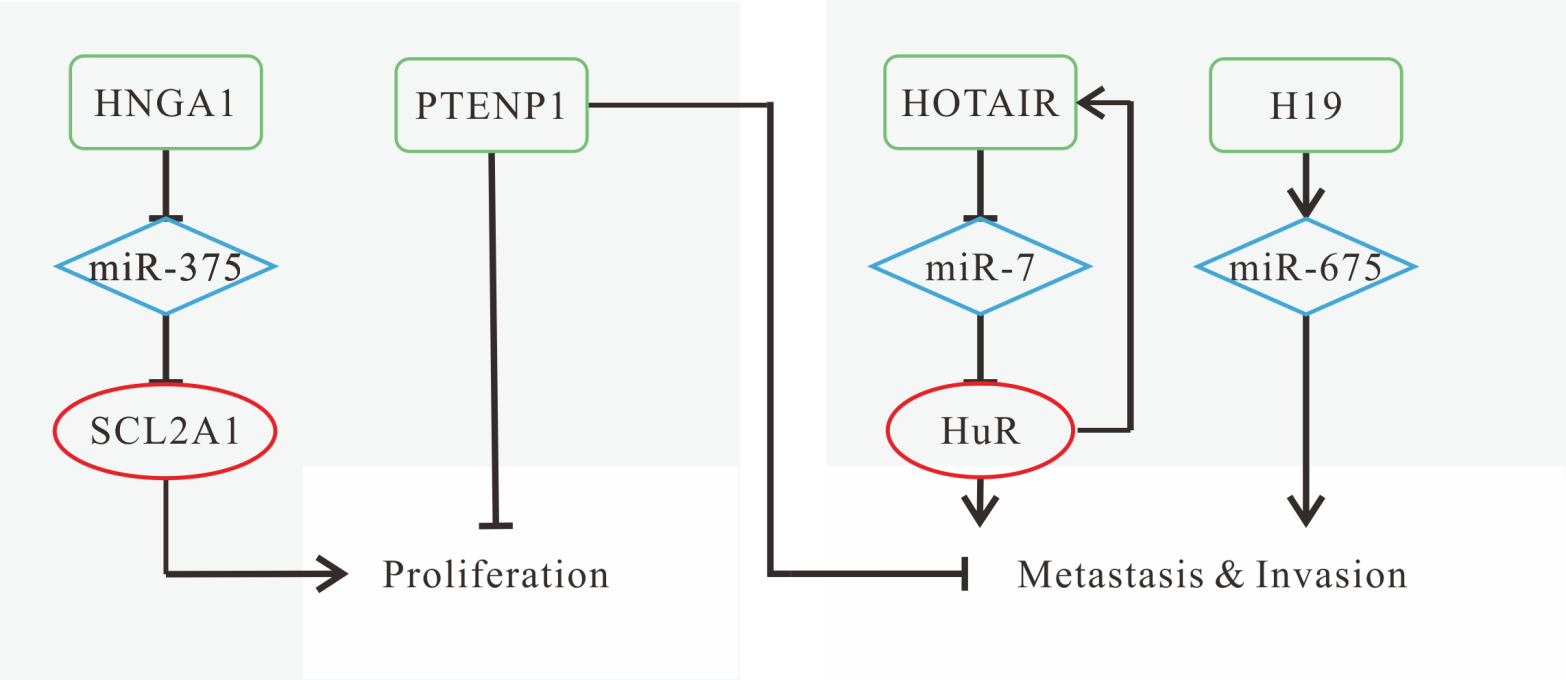
Mechanisms of lncRNAs in HNSCC progression (**A**) HNGA1 promotes cell proliferation and glycolysis by acting as a ceRNA of miR-375 to increase SCL2A1 levels; (**B**) down-regulated PTENP1 promotes growth, proliferation, colony formation, migration and invasion; (**C**) HOTAIR promotes metastasis by forming a feed-forward regulatory loop with HuR; (**D**) H19 promotes cell viability, migration and invasion by miR-675 (the mature product of H19) overexpression.

HNSCC ranked the sixth among the most common cancers with only 40% five-year-survival and nearly 60% mortality within five years because of high metastases and recurrences [62]. Research has focused on therapies, and lncRNAs can be promising strategies to inhibit cancer progression. The known mechanisms of HNSCC-related lncRNAs are shown in Figure 1. The knockdown of HOTAIR, H19/miR-675 and HNGA1 leads to poor proliferation, migration and invasion [42, 43, 51, 52], and overexpression of PTENP1 could inhibit cell proliferation, invasion and colony formation [55]. Individually, HOTAIR depletion induces mitochondrial calcium uptake 1-dependent cell death [43].

LncRNAs can also be adapted to diagnosis and prognosis of HNSCC. Higher expression levels of HOTAIR, H19, HNGA1 are observed in HNSCC compared with normal tissues [43, 51, 52], while PTENP1 is down-regulated [55], which is believed to be promising biomarkers for diagnosis besides conventional diagnostic method. To inhibit tumor growth and migration, the expression levels of specific lncRNAs are adjusted to the opposite sides, and the alterations achieve willing effect. For prognosis, highly expressed HOTAIR, H19 and HNGA1 or down-regulated PTENP1 are risky factors. Patients with high expression of HOTAIR, H19 or low expression of PTENP1 have poorer OS and disease-free survival (DFS) [43, 51, 52, 55, 63]. Highly expressed HOTAIR is correlated with tumor size and lymph node metastasis [43], while H19 can contribute to higher patient relapse [51, 63]. HNGA1 is correlated with post-operative survival [52]. Besides, the methylation status of PITX2 gene and its adjacent lncRNA PANCR are convinced to be effective predictors for OS [61].

### NPC-associated lncRNAs

#### HOTAIR

HOTAIR expression is significantly increased in NPC tissues [64, 65]. In invasive NPC cell lines, the expression level of HOTAIR is up-regulated compared with that in high-differentiated cell lines [64]. HOTAIR can facilitate NPC tumorigenesis by functioning as an angiogenic inducer and promoting cell proliferation through direct and indirect signaling pathways: activating vascular endothelial growth factor-A (VEGFA) transcription by directly targeting its promoter, or up-regulating VEGFA and Ang2 by increasing glucose-regulated protein 78 (GRP78) expression [65]. Knockdown of HOTAIR leads to inhibition of cell proliferation, migration and invasion as well as stimulation of cell apoptosis [65].

#### H19

H19 upregulation was observed in the poor-differentiated NPC cell line compared with the normal nasopharyngeal epithelial cell line [66]. Hypermethylation of the CpG locus in the H19 promoter region can lead to the imbalance of H19 gene expression, suggesting that hypermethylation of the gene promoter region may involve in the differentiation of human NPC cells and transcriptional silencing of imprinted genes [66]. Furthermore, H19 inhibits E-cadherin expression, induces Epithelial-Mesenchymal Transition (EMT), and promotes invasion in NPC by modulating the miR-630/ enhancer of zeste homolog 2 (EZH2) axis [67]. EZH2, a downstream target of miR-630, can be down-regulated by miR-630. The elevated H19 represses the activity of miR-630, thus increasing EZH2 expression. EZH2 accumulation induces the silence of epigenetic target genes, and promotes cell invasion [67].

#### MALAT1

Metastasis-associated lung adenocarcinoma transcript 1 (MALAT1), a long non-coding RNA transcribed from gene locus on chromosome 11q13, is reported to be associated with a variety of human tumors, such as lung, gastric and bladder cancer [68–70]. Recently, emerging evidence has demonstrated that aberrantly expressed MALAT1 involves in the tumorigenesis and tumor progression in HNC. MALAT1 accumulation can enhance proliferative capacity, metastatic and invasive potential, as well as induce apoptosis in the most common types of HNC, like thyroid carcinoma, NPC, LSCC, TSCC and OSCC [71–79]. MALAT1 is up-regulated in NPC. Several studies have elaborated on the correlation and the possible interactive molecular mechanisms between MALAT1 and HNC. Two molecular effects of MALAT1, either regulating alternative splicing or transcription, have been uncovered based on a sequence of experiments [80]. MALAT1, as a ceRNA, promotes radioresistance of NPC cells via modulating miR-1/slug axis [72]. MALAT1- induced downregulation of miR-1 elevates Slug protein, a downstream target of miR-1, and then increases cancer stem cell (CSC) activity and radioresistance of NPC cells. Over-expressed miR-1 can decrease MALAT1 expression in turn, indicating that there might be a reciprocal repression loop between MALAT1 and miR-1 [72].

Collectively, MALAT1 might serve as a promising diagnostic, prognostic biomarker and therapeutic target for HNC patients. We infer that resveratrol, which is of potential use in prevention and treatment of colorectal cancer [81], might also have the same anti-tumor effect on HNC through the inhibition of Wnt/β-catenin signaling pathway.

#### ANRIL

ANRIL (antisense noncoding RNA in the INK4 locus), is a long noncoding RNA spanning 126.3kb at the antisense orientation of the INK4B-ARF-INK4A gene cluster, whose spliced product is a 3834bp RNA consisting of 19 exons. ANRIL has been reported to be related to epigenetic transcriptional repression of the INK4 locus through the interactions between Chromobox 7 (CBX7) and ANRIL [82]. Accumulating evidence has proved that ANRIL has the oncogenic ability to induce NPC. Highly expressed ANRIL could enhance cell proliferative and transforming capacity, as well as increase the proportion of side population cells (SP cells) in NPC [83]. ANRIL could also reprogram glucose metabolism for energy provision of cell proliferation. ANRIL-mediated up-regulation of Glut1 and LDHA, associated with the regulation of basal uptake of glucose and aerobic glycolysis, respectively, can enhance glucose uptake for rapid ATP production in NPC cells. We infer that ANRIL might activate the mTOR signal pathway via promoting the phosphorylation of Akt, and thus influences the expression of essential genes in glycolysis [83]. Additionally, miRNA let-7a is down-regulated in NPC tissues and negatively correlated with ANRIL expression. ANRIL knockdown might reduce carcinogenic ability and increase DDP-induced cytotoxicity by increasing miR-let-7a expression in NPC [84].

#### ROR

LncRNA-ROR, consisting of four exons, is located at chromosome 18q21.31 [85]. As a regulatory molecule, increasing evidence has presented that lncRNA-ROR is associated with tumorigenesis, progression and metastasis in multiple tumors. A recent study has reported lncRNA-ROR is significantly up-regulated in human NPC tissues [86]. Overexpressed lncRNA-ROR decreases the apoptosis rate and promotes cell proliferation. LncRNA-ROR can promote cell metastatic and invasive ability by inducing an EMT phenotype in NPC cells, indicating that ROR may serve as an oncogene [86]. Moreover, the regulation of p53 and p21 at the translational level after chemotherapy also contributes to chemoresistance. Highly expressed lncRNA-ROR may enhance the chemotherapy resistance ability of NPC cells by inhibiting the p53 pathway [86].

Previous research has demonstrated that EMT of tumor cells not only increases migration, but also leads to drug resistance [87]. Down-regulated lncRNA-ROR in breast cancer cells can inhibit the EMT process and cell invasion, and increase the sensibility to tamoxifen [88]. Based on these findings, we infer that lncRNA-ROR does play a critical role in NPC progression. Further investigation is required to explore the specific mechanisms about how lncRNA-ROR contributes to NPC migration and chemotherapy resistance.

#### AFAP1-AS1

Actin filament associated protein 1 antisense RNA1 (AFAP1-AS1), has been confirmed to be associated with metastasis and poor prognosis of NPC in recent years [89]. AFAP1-AS1 affects the expression of cytoskeletally-regulated proteins via inhibiting the Rho/Rac signaling pathway in NPC. Knockdown of AFAP1-AS1 significantly increases expression of small GTPases such as Rhogdi, Pfn1, Rab10, Rab11a, Rac2 and RhoA in the pathway while decreases RhoC, Rab11b and Lasp1 proteins. Furthermore, AFAP1-AS1 can promote NPC cell migration by regulating the integrity of actin filament [89]. The same molecular mechanism has been observed in lung cancer [90] and hepatocellular carcinoma [91].

#### LET

The expression level of lncRNA-LET has been found significantly lower in NPC tissues than in paired normal tissues, and the down-regulated LET is related to larger tumor size and poor prognosis of NPC patients. *In vivo* experiment shows that enhanced LET expression inhibits cell proliferation and induces cell apoptosis by up-regulating the expression level of cleaved Caspase-3. The attenuated LET expression is induced by EZH2-mediated H3K27 histone methylation in the LET promoter region. Elevated LET expression represses cell proliferation and induces apoptosis of NPC cells, while knockdown of LET could reverse the effects [92]. Collectively, the inverse correlation between the expression of lncRNA-LET and EZH2 may provide a potential therapeutic method for NPC treatment.

#### LINC0086

LncRNA LINC0086 is found down-regulated in serum samples and tissues of NPC patients, and the Kaplan-Meier survival curve shows that the expression level of LINC0086 is positively correlated with the survival rate of NPC patients. MiR-214 is widely recognized as an oncogene in malignant tumors, and miR-214 expression could be reduced through directly interacting with LINC0086. Based on these discoveries, it is suggested that overexpression of LINC0086 could inhibit cell proliferation and promote apoptosis in NPC through the repression of miR-214 expression. Interestingly, the inhibitory effects of LINC0086 on NPC cells could also be reversed by miR-214 overexpression [93].

#### LOC401317

LncRNA LOC401317 expression has been reported significantly up-regulated along with TP53 overexpression in NPC cell line HNE2, and LOC401317 is directly modulated by p53 at the transcriptional level. Overexpression of LOC401317 triggers HNE2 cell cycle arrest at the G0/G1 phase, as well as increases the apoptosis rate. Interestingly, LOC401317 doesn’t form a feedback loop with either p53 or MDM2, and LOC401317 expression has no effect on them. LOC401317 alters the expression levels of several key effector molecules of cell cycle progression and apoptosis. Elevated expression of LOC401317 might result in the trigger of cell cycle arrest through increased p21 and decreased cyclin D1 and E1, as well as the promotion of cell apoptosis by activating poly (ADP-ribose) polymerase (PARP) and caspase 3 [94].

#### Application

NPC is a group of malignant lesions, the symptoms of which vary from non-specific ones to cranial nerve palsies [1]. The incidence of NPC has gradually decreased over the past few decades. However, the early diagnosis and treatment strategies remain to be improved [95].

In NPC tissues, HOTAIR, H19, MALAT1, ANRIL, ROR and AFAP1-AS1 are up-regulated [64, 67, 72, 83, 86, 89], while LET, LINC0086, LOC401317 and LINC00312 are down-regulated [92–94, 96], which is correlated with their applications in treatment and prognosis. Both knockdown of HOTAIR, H19, MALAT1, ANRIL, ROR and AFAP1-AS1, and overexpression of LET, LINC0086 and LINC00312 may have inhibitory effects on cell viability, migration and invasion [64, 65, 67, 84, 86, 89, 92–94, 97]. Moreover, knockdown of MALAT1 can increase radiation sensitivity [78]. The inhibition of ROR expression can reduce chemotherapy resistance [86].

**Figure 2:**
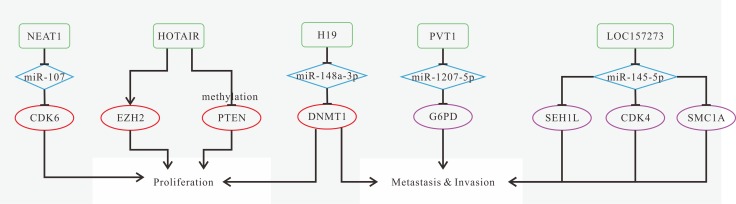
Mechanisms of lncRNAs in NPC progression (**A**) HOTAIR promotes cell growth and angiogenesis by inducing VEGFA expression directly or indirectly; (**B**) MALAT1 promotes cancer stem cell activity and induces radioresistance to up-regulate slug by reducing miR-1 activity; (**C**) ANRIL enhances the proliferative and transforming capacity, reprograms cell glucose metabolism and induces SP cells possibly through activation of the mTOR signal pathway; d. low LET expression is induced by EZH2-mediated H3K27 histone methylation in the LET promoter region; (**D**) down-regulated LINC0086 promotes proliferation and inhibits apoptosis by decreasing the expression of miR-214; (**E**) down-regulated lncRNA LOC401317 promotes NPC cell cycle progression by down-regulating p21 and up-regulating cyclin D1 and E1, as well as inhibits apoptosis by inhibiting PARP and caspase 3; (**F**) ROR promotes metastasis and invasion by inducing EMT; (**G**) H19 induces EMT and promotes migration and invasion via modulating the miR-630/EZH2 axis; (**H**) AFAP1-AS1 promotes metastasis by increasing expression of AFAP1 and some cytoskeleton-regulated proteins.

For prognosis, highly expressed HOTAIR is the indication of poor local recurrence-free survival, OS and DFS [64]. High expression of MALAT1 causes poor overall survival [72], while high expression of ANRIL has longer OS and shorter DFS [83]. Highly expressed AFAP1-AS is correlated with distant tumor metastasis, and indicates poorer OS and relapse-free survival [89]. Down-regulated LET is correlated with tumor size and poorer prognosis [92]. LINC0086 and LINC00312 expression levels are associated with lymph node metastasis, and patients with higher expression are predicted to have a higher survival rate [93, 96]. The known mechanisms of NPC-related lncRNAs are shown in Figure 2.

### TC-associated lncRNAs

#### BANCR

Recently, studies have found a novel lncRNA, BRAF-activated non-coding RNA (BANCR), is up-regulated in PTC tissues and PTC IHH-4 cells compared with adjacent normal tissues [98, 99]. The reduced expression of BANCR in IHH-4 cells can significantly inhibit cell proliferation and induce apoptosis, but has no effect on cell migration [98]. Enhanced BANCR expression in PTC may promote cell proliferation by decreasing the percentage of cells in G1 phase, and inhibit apoptosis through activation of autophagy [98]. Furthermore, knockdown of BANCR inhibits tumor cell growth and induces cell cycle arrest at G0/G1 phase through the reduction of cyclin D1 [99]. Highly expressed BANCR may increase the expression level of thyroid-stimulating hormone receptor (TSHR) via increasing EZH2 recruitment in PTC, which may be one of the possible mechanisms that BANCR contributes to PTC [99].

Interestingly, BANCR can serve as either a carcinogenic factor or a tumor suppressor, and several studies have reported completely different results from the above findings. The expression of BANCR has been found down-regulated in PTC tissues and PTC cell lines (TPC-1, K1, and BCPAP) [100]. And upregulation of BANCR in K1 cell lines inhibits cell proliferation and invasion, as well as induces apoptosis [100]. BANCR plays a significant role in inactivation of the MAPK pathway in tumors, such as malignant melanoma [101] and lung cancer [102]. Highly expressed BANCR can affect MAPK pathway by reducing the expression levels of phosphorylated ERK and phosphorylated p38 in K1 cell lines [100].

Collectively, BANCR expression level differs from PTC cell lines. Highly expressed BANCR can promote the PTC development by modulating cell cycle, autophagy activation or the expression level of TSHR, while down-regulated BANCR can exert its effect by affecting the MAPK pathway. We suggest that there might be other unknown factors involved in regulating the expression level of BANCR in PTC, and the specific mechanisms require further investigation.

#### PTCSC

Recently, a class of long intergenic noncoding RNAs named papillary thyroid cancer susceptibility candidate (PTCSC), such as PTCSC1 (located in 8q24), PTCSC2 (located in 9q22) and PTCSC3 (located in 14q13), might predispose infected persons to thyroid cancer. The expression levels of PTCSC2 and PTCSC3 are strictly thyroid-specific, and they may serve as promising tumor suppressors [103, 104].

PTCSC2 is significantly down-regulated in PTC tissues compared with paired normal tissues [103]. PTCSC2 may enrich the expression of other genes involved in cell cycle and cancer progression. Significant correlation was observed between the risk allele [A] of single nucleotide polymorphism (SNP) rs965513 and the low expression levels of *PTCSC2, FOXE1,* and TSHR in non-tumor thyroid tissues along with high PTC risk, suggesting that there might exist a multilayer regulatory network. Additionally, the 5’ end of PTCSC2 (isoform c) overlaps the promoter region of FOXE1. Myosin-9 (MYH9), as a binding protein of PTCSC2, can regulate the transcription of FOXE1 and PTCSC2 via inhibiting the activity of FOXE1-and-PTCSC2-shared promoter, while PTCSC2 overexpression can reverse MYH9-induced promoter inhibition [105].

PTCSC3 expression is also significantly decreased in PTC tissues, and SNP rs944289 risk allele [T] is related to the downregulation of PTCSC3. SNP rs944289 overlaps the binding site for CCAAT/enhancer binding protein (C/EBP) α and β, and either C/EBPα or C/EBPβ can activate the PTCSC3 promoter. PTCSC3 can influence the expression of genes associated with DNA replication, recombination and repair, tumor cell viability and motility and tumor morphology [104]. Moreover, PTCSC3 inhibits cell growth, promotes apoptosis and arrests cell cycle at G1/S and G2/M phases in thyroid cancer [106]. Overexpression of PTCSC3 can reduce the expression of miR-574-5p by acting as a ceRNA for miR-574-5p in different thyroid cancer types (of papillary, follicular and anaplastic origin) [106]. These findings suggest that PTCSC3 might regulate cell growth and apoptosis by targeting miR-574-5p.

Generally, the risk allele may involve in the reduction of PTCSC2 and PTCSC3 expression and the increased risk of thyroid cancer. Both PTCSC2 and PTCSC3 can play an important anti-tumor role by affecting the expression levels of other genes in thyroid cancer, but the specific mechanisms require further investigation.

#### PVT1

LncRNA PVT1 is transcribed from a gene located on chromosome 8q24, and the transcription can be regulated by Myc [107]. LncRNA PVT1 has been found significantly up-regulated in thyroid tissues. PVT1 silencing can repress proliferation, induce G0/G1 cell cycle arrest and decrease the expression of cell cycle-related protein cyclin D1 and TSHR expressions at mRNA and protein levels in three kinds of TC cell lines IHH-4 (PTC), FTC-133 (FTC), and 8505C (ATC) [108]. Moreover, PVT1 knockdown reduces EZH2 recruitment. Silencing of PVT1 may down-regulate TSHR by binding to EZH2, and lead to proliferative inhibition in TC cells which are mediated by the interactions between TSH and TSHR. In all, the interactions between PVT1 and EZH2 present the oncogenic ability in TC progression [108].

#### NEAT1

LncRNA Nuclear Enriched Abundant Transcript 1 (NEAT1), also named as Nuclear Paraspeckle Assembly Transcript 1, is transcribed from the multiple endocrine neoplasia locus on chromosome 11, and it is confirmed to play a crucial role in the structure of paraspeckles [109, 110]. Previous research has revealed NEAT1 is associated with multiple human cancers, including prostate cancer, hepatocellular carcinoma, glioma and acute promyelocytic leukemia cells [111–114].

NEAT1 and Arg-1 are highly expressed in PTC while miR-214 is down-regulated, which promotes the development and progression of PTC [115]. NEAT1 down-regulates miR-214, and then increases the expression of β-catenin and promotes PTC malignant behaviors like migration and invasion [115]. Based on the reciprocal repression correlation between NEAT1 and miR-214, we can infer that upregulation of miR-214 might inhibit PTC malignant progression through the reduction of NEAT1 expression, offering a promising option for PTC treatment.

#### FAL1

Focally amplified lncRNA on chromosome 1 (FAL1), is transcribed from a focal amplicon on chromosome 1q21.2. Previous research has revealed that FAL1 can reduce the expression of cyclin-dependent kinase inhibitor 1A (CDKN1A, p21) via binding to the BMI1 proto-oncogene (BMI1), thus increasing cyclin-dependent kinase (CDK) activity, phosphorylating retinoblastoma protein (RB), inducing E2F transactivation, and finally promoting the G1/S transition [116]. However, p21 mRNA expression is increased in PTC, contrary to the decrease in other cancers, indicating that p21 expression is not correlated with FAL1’s in PTC. Also, FAL1 overexpression in PTC can lead to a significant increase in cyclin D1, E2F1, E2F2 and VEGF-A, suggesting FAL1 plays an oncogenic role in promoting proliferation and generating aggressive behavior such as multifocality in PTC [117].

#### GAS8-AS1

LncRNA growth arrest-specific 8-antisense RNA 1 (GAS8-AS1) has been identified as the secondary most frequently mutated gene (9.2%), supported by the analysis of 402 Chinese PTC samples. LncRNA GAS8-AS1 is down-regulated in PTC tissues compared with paired non-tumor tissues. Ectopic expression of both wild-type and mutated lncRNA GAS8-AS1 in PTC cell lines can inhibit cell viability, and the wild-type shows a more remarkable inhibitory effect than the mutated one. Among the 8 patients of 91 PTC cases with GAS8-AS1 somatic mutations, seven were found to carry a c.713A>G/714T>C dinucleotide substitution, which might contribute to a higher rate of dismal prognosis [118]. Based on the findings, lncRNA GAS8-AS1 might function as a tumor suppressor, as well as a potential diagnostic and therapeutic target.

#### NAMA

BRAF mutation is one of the most common genetic alterations in PTC, and it is closely related to PTC development by influencing the MAP kinase signaling pathway. It has been reported a new lncRNA, noncoding RNA associated with mitogen-activated protein kinase pathway and growth arrest (NAMA), is down-regulated in PTC [99, 119]. The reduction of BRAF expression, inactivation of MAP pathway or DNA damage could trigger NAMA expression, and then induce cell cycle arrest [119]. Each of these 3 alterations can activate Raf/MEK/ERK signaling pathway in PTC, which is related to cell growth, proliferation and differentiation [119, 120]. Recent studies have demonstrated that there is no statistically significant difference between NAMA and TSHR expression in PTC [99]. NAMA is a promising target for future PTC treatment, but further research is needed to reveal the specific mechanisms of its function.

#### HOTAIR

HOTAIR was significantly up-regulated in TC compared with adjacent tissues. The results were similar to those detected in TC patients’ plasma, whereas there was almost no HOTAIR expression in the plasma of the healthy volunteers [121]. HOTAIR susceptibility SNP rs920778 genetic variants (such as TT, TC and CC genotype) are significantly associated with PTC initiation via engaging in the regulation of HOTAIR expression. Subjects with the TT or CT genotype have higher HOTAIR RNA levels in non-tumor tissues and also higher risk of PTC developing than those with the CC genotype [122].

#### MALAT1

MALAT1 has been reported highly expressed in both non-cancerous thyroid tissues and PTC tissues, with increasing expression during PTC progression, whereas it is down-regulated in ATCs and PDTCs [71]. EMT is now identified as a process related to cell migratory and invasive properties, with the loss of cell polarity and adhesion. TGF-β-induced EMT can increase MALAT1 expression in PTC, suggesting it might also involve in the pathogenesis of thyroid malignancies [71]. IQ motif containing GTPase activating protein 1 (IQGAP1), is a scaffold protein involving in cellular processes via binding to cell adhesion, cytoskeletal and signal transduction proteins, like Rac1 and Cdc42. It has also been revealed that enhanced MALAT1 promotes cell proliferation and invasion in FTC by increasing IQGAP1 expression, while IQGAP1 knockdown can reverse the effect of MALAT1 [76].

#### Application

According to a survey in the United States, after years of research, the diagnosis of TC has increased a lot, but the morality of TC remains stable [123]. Papillary thyroid cancer, the most common type of TC, has shown a sharp rise in incidence [124]. Besides, TC is difficult to recognize, and more specific methods need to be investigated.

**Figure 3:**
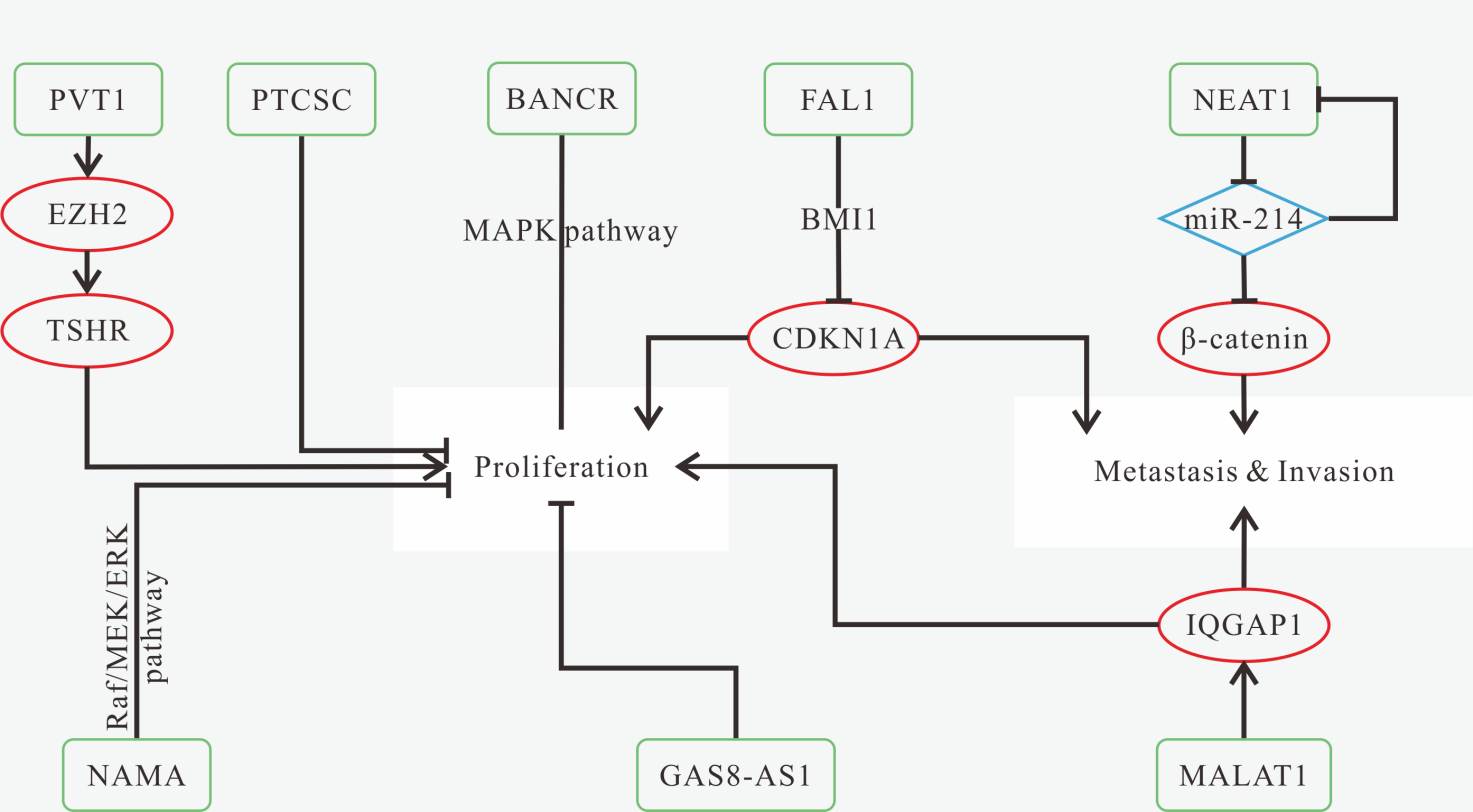
Mechanisms of lncRNAs in TC progression (**A**) silencing of PVT1 represses proliferation and decreases cyclin D1 expression through reducing recruitment of EZH2 and modulating TSHR; (**B**) down-regulated PTCSC3 promotes cell growth and inhibits apoptosis; (**C**) BANCR overexpression inhibits cell proliferation and invasion, and induces apoptosis through inactivation of ERK and p38 in PTC cell lines (TPC-1, K1, and BCPAP); (**D**) FAL1 can reduce the expression of CDKN1A via binding to BMI1, thus promoting proliferation and migration; (**E**) NEAT1 promotes migration and invasion, as well as increases the expression of β-catenin through the reduction of miR-214; (**F**) downregulation of NAMA inhibits apoptosis via modulating Raf- MEK-ERK signaling pathway; (**G**) down-regulated GAS8-AS1 promotes cell viability; (**H**) MALAT1 promotes proliferation and invasion by up-regulating the expression of IQGAP1 in FTC.

Previous research has found that the expression levels of FAL1, HOTAIR, LOC100507661, NEAT1, NONHSAT076754 are increased [117, 122, 125–127], while GASS-AS1, NAMA, NONHSAT037832 are decreased in tumor tissues [118, 119, 128], which could be indicators for diagnosis. The known mechanisms of TC-related lncRNAs are shown in Figure 3. Knockdown or depletion of HOTAIR, LOC100507661, NEAT1 as well as overexpression of NAMA can inhibit tumor progression [119, 122, 126, 127]. Knockdown of NONHSAT076754 can put an inhibitory effect on cell invasion and lymph node metastasis [125]. These findings suggest promising therapeutic strategies for TC patients. FAL1, NONHSAT037832 and NONHSAT076754 could be biomarkers for prognosis [117, 125, 128]. Highly expressed FAL1 increases the risk of multifocality [117]. NONHSAT037832 and NONHSAT076754 are associated with lymph node metastasis [125, 128]. Interestingly, BANCR expression exhibits an opposite trend according to different PTC cell lines. It is up-regulated in PTC cell line IHH-4 [98, 99], while down-regulated in cell lines TOC-1, K1 and BCPAP [100].

### TSCC-associated lncRNAs

#### MALAT1

Additionally, it is verified that the interaction between MALAT1 and miR-124 also plays a critical part in tongue cancer cell growth [73], and miR-124 has been previously confirmed to be associated with HR-HPV-positive cervical cancer and breast cancer [129, 130]. Overexpression of MALAT1 could reduce miR-124 expression, and subsequently facilitate the growth and metastasis of tongue cancer cells via targeting jagged1 (JAG1), a downstream gene of miR-124. The expression level of JAG1 is positively correlated with MALAT1 but negatively correlated with miR-124 expression, and the elevated JAG1 is essential for MALAT1-mediated cell growth [73].

Overexpression of MALAT1 also induces EMT, promotes migration and invasion, as well as inhibits apoptosis of TSCC cells through the activation of Wnt/β-catenin signaling pathway [74]. Besides, Dickkopf-related protein 1 (DKK1), a repressor of Wnt signaling pathway, has been found effectively eliminated the effects of exogenous MALAT1 on vimentin (up-regulated) and E-cadherin (down-regulated), providing a promising therapeutic strategy for TSCC treatment [74]. Recent studies have shed new light on the participation of lncRNAs in tumor initiation and development by modulating protein-coding genes. Knockdown of MALAT-1 leads to the increased expression of several members of small proline-rich proteins (SPRR) in TSCC cells, and high expression levels of certain SPRR proteins (such as SPRR2A) can inhibit the distant metastatic potential of TSCC cells *in vivo* [75].

#### MEG3

Maternally Expressed Gene 3 (MEG3) is an imprinted gene located at chromosome 14q32 and encodes a lncRNA which is pervasively expressed in normal tissues and confirmed as a tumor suppressor. However, loss of MEG3 expression has been observed in multiple malignant cancers, including breast cancer, hepatocellular cancer and TSCC [131–133], and MEG3 silencing in cancer cells is mainly associated with its promoter hypermethylation [134]. Moreover, re-expression of MEG3 can inhibit tumor progression by inducing p53 expression, a tumor suppressor. The expression of p53 is very low in normal tissues due to the rapid ubiquitin-proteasome-mediated degradation, while re-expression of MEG3 can stimulate p53 transcription, reduce its degradation and increase its protein levels in tumor cells [134].

Recently, the expression levels of MEG3 and miR-26a have been found significantly decreased in TSCC tissues and correlated with TSCC progression [133]. MiR-26a has been known to inhibit DNA methyltransferase 3B (DNMT3B) transcripts. Also, experiments have demonstrated that miR-26a can increase the expression of unmethylated MEG3, suggesting that MEG3 can be up-regulated by the inhibitory effect of miR-26a on DNMT3B transcripts. Furthermore, MEG3 accumulation inhibits cell proliferation and causes cell cycle arrest and apoptosis of TSCC cell lines (SCC-15 and CAL27) [133]. The same mechanism has also been reported in hepatocellular carcinoma, and miR-26a regulates cell proliferation and migration by modulating DNMT3B/MEG3 axis [135]. Patients with low MEG3 expression have statistically shorter OS than those with high MEG3 expression, suggesting MEG3 can function as a tumor suppressor and independent predictive factor for TSCC patients [133].

#### NKILA

Nuclear Factor-κB Interacting LncRNA (NKILA), encoded by the gene located on chromosome 20q13, has been reported to be down-regulated in TSCC tissues and the tissue samples with high tumor node metastasis (TNM) stage. *In vitro* experiments have demonstrated that NKILA expression is negatively correlated with TSCC metastasis [136].

Aberrant NF-κB activation affects the occurrence and development of multiple cancers [137]. NF-κB is released for DNA binding and transcriptional activation by IκB kinase (IKK)-induced phosphorylation of IκB [138], and the increased NF-κB activity leads to the enhancement of tumor cell invasion ability. In the previous study of breast cancer [139], NKILA transcription was induced by NF-κB signaling pathway, while NKILA expression and NF-κB activity were negatively correlated. NKILA can inhibit NF-κB activation by suppressing IKK-induced IĸBα phosphorylation but not IKK activity, resulting in the repression of tumor cell migration and invasion. NKILA associates with the NF-κB: IκBα complex via binding to p65, and thus forms a stable NF-κB: IκBα: NKILA complex. The formation of NF-κB: IκBα: NKILA complex is the core of the whole process. The molecular mechanism has also been confirmed in TSCC [136], breast cancer [139] and melanoma [140].

NF-κB plays a pivotal role in regulating tumor cell EMT. The down-regulated NKILA can significantly decrease E-cadherin expression, while increase the expression of N-cadherin, twist and vimentin. Moreover, NKILA regulates cells migration by modulating NF-κB/Twist pathway. These results indicate that the reduction of NKILA in TSCC can eliminate the inhibitory effect on NF-κB, and thereby promote the EMT process as well as migration and invasion in TSCC [136].

Additionally, low NKILA expression is significantly correlated with advanced clinical stage, tumor size and lymph node metastasis in TSCC [136]. In conclusion, NKILA can affect TSCC progression and metastasis, and function as a prognostic biomarker for TSCC.

#### UCA1

Urothelial cancer-associated 1 (UCA1) was first thought to participate in bladder cancer invasion and progression. It is one of the lncRNAs correlated with tumor lymph node metastasis. The expression level of UCA1 in TSCC cells with lymph node metastasis (LNM) is significantly higher than those without LNM. Compared with primary tumors, the increased UCA1 expression in lymph node metastasis indicates that overexpression of UCA1 can enhance cell migration in TSCC. Moreover, it is reported that the elevated UCA1 has little effect on TSCC cell proliferation [141]. Until then, the specific mechanisms about how UCA1 contributes to TSCC remain unclear.

#### LINC00673

Compared to adjacent non-tumor tissues, lncRNA LINC00673 is highly expressed in TSCC tissues. High LINC00673 expression was positively correlated with tumor size, invasion muscles of tongue and higher TNM stage [142]. Knockdown of LINC00673 inhibits the *migration* and invasive properties of TSCC cell lines (Tca8113 and Cal27). Up-regulated LINC00673 is positively correlated with tumor size, higher TNM stage and relapse. Additionally, the expression level of LINC00673 is negatively associated with OS and relapse-free survival [142]. In summary, LINC00673 can be regarded as a regulator of TSCC progression, as well as prognostic predictor of TSCC.

#### Application

OSCC is one of the squamous cell carcinomas, and the prognosis of young people is reported to be poor and aggressive [143]. Biomarkers to detect TSCC have been reported, but the conclusions are not so reliable [144].

**Figure 4:**
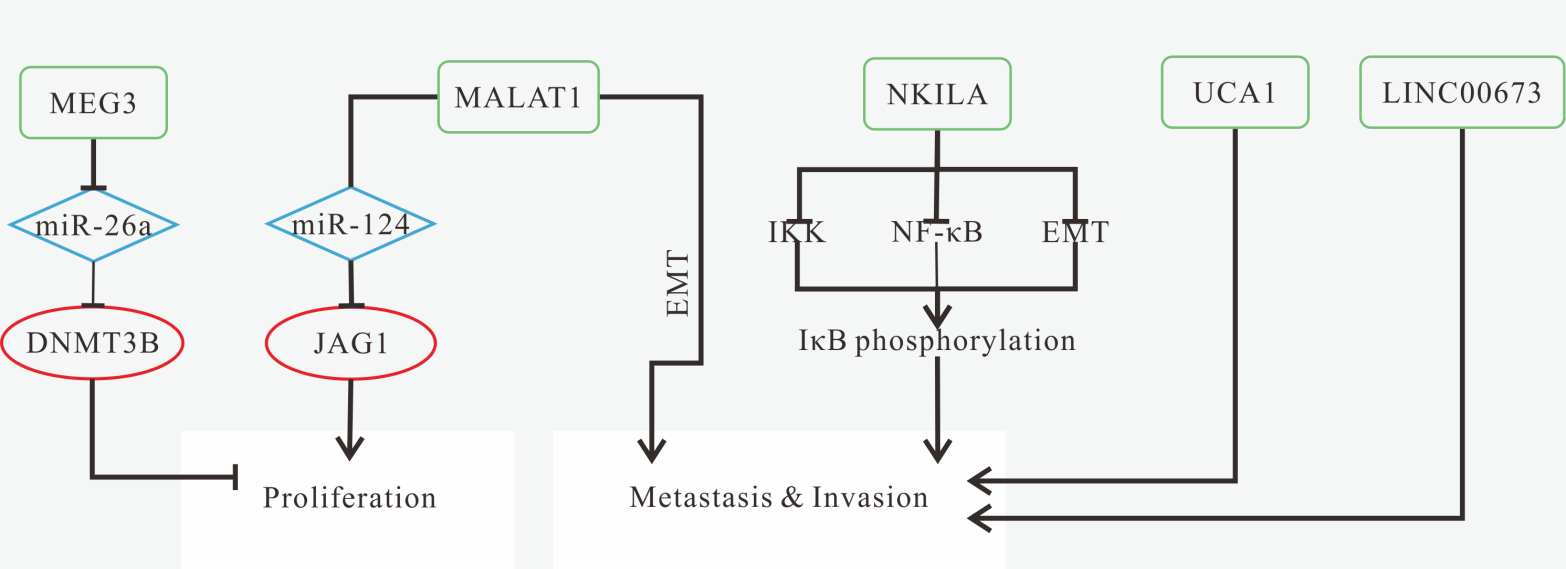
Mechanisms of lncRNAs in TSCC progression (**A**) down-regulated MEG3 results in miR-26a overexpression, and then reduce DNMT3B expression, thus promoting cell proliferation and inhibiting apoptosis; (**B**) MALAT1 overexpression promotes tumor growth and invasion by increasing JAG1 expression resulted from the reduction of miR-124; induces EMT; (**C**) down-regulated NKILA promotes tumor cell migration and invasion via promoting IκBα phosphorylation induced by IKK, NF-κB activation and the induction of EMT process; (**D**) UCA1 promotes migration; (**E**) LINC00673 promotes migration and invasion.

Either high expression of HOTTIP, LINC00152, LINC00673, MALAT1 and UCA1, or low expression of AC007392.4, MEG3 and NKILA is found in tumor tissues [75, 133, 136, 141, 142, 145, 146]. The known mechanisms of TSCC-related lncRNAs are shown in Figure 4. Knockdown of LINC00673, MALAT1 and UCA1, and overexpression of MEG3 and NKILA can inhibit tumor progression, which might become prospective treatment methods [133, 136, 141]. Patients with high expression of HOTTIP, LINC00152 and LINC00673 as well as low expression of MEG3 and NKILA have poorer OS [133, 142, 145, 146]. MALAT1, NKILA and UCA1 are associated with lymph node metastasis [75, 136, 141].

### OSCC-associated lncRNAs

#### HOTAIR

Mechanistically, HOTAIR induces gene silencing through interactions with histone methyltransferase polycomb repressive complex 2 (PRC2) and histone demethylase lysine-specific demethylase 1 (LSD1). HOTAIR recruits the PRC2 and LSD1/ CoREST (REST corepressor 1) / REST (RE1-silencing transcription factor) complexes to target gene, and represses gene transcription via reprograming chromatin states caused by PRC2-mediated histone H3 lysine 27 (H3K27) tri-methylation and LSD1-mediated histone H3 lysine 4 (H3K4) demethylation [147].

In OSCC, HOTAIR interference induces cell cycle arrest in the G0/G1 phase and apoptosis [148]. As mentioned above, HOTAIR involves in epigenetic modification, and E-cadherin expression can be reduced partly by enrichment of EZH2 [149]. HOTAIR accumulation down-regulates E-cadherin expression via recruiting EZH2 and H3K27me3 to the E-cadherin promoter, and thus increases OSCC cell motility, favoring tumor cell migration and invasion [149]. These results suggest that HOTAIR might be related to the inducement of EMT in OSCC cells.

#### PTENP1

PTENP1, a ceRNA, can protect PTEN transcripts from being degraded by PTEN-targeting miRNAs. PTENP1 can increase PTEN expression in OSCC cell lines via competitively binding to miR-21, leading to the inhibition of proliferation and colony formation [150]. Furthermore, cells at S phase has increasing trends while cells at G2/M phase has decreasing trends due to overexpression of either PTEN or PTENP1. PTENP1 overexpression induces cell cycle arrest at S-G2/M phase via restraining the AKT pathway in OSCC [150]. Previously, it has been verified that loss of PTEN activity resulted in PIP3 accumulation, which could have the same effect as PI3K on activating its downstream effector AKT, and then the activated AKT contributed to malignant tumors [151]. In conclusion, both PTENP1 and PTEN can serve as tumor suppressors and reduces HNSCC tumorigenicity.

#### UCA1

Research has found UCA1 affects OSCC through different mechanisms [152]. UCA1 overexpression is positively correlated with the activation of Wnt/β-catenin signaling pathway. UCA1 silencing in OSCC cells could repress proliferation, metastasis and invasion, as well as induce apoptosis via modulating the Wnt/β-catenin signaling pathway and the expression of downstream genes. Knockdown of UCA1 inhibits tumor growth *in vivo*, suggesting that UCA1 may provide a lncRNA-oriented diagnostic and therapeutic strategy for OSCC [152]. The similar mechanism was also found in breast cancer [153].

UCA1 has also been reported to be associated with drug resistance in breast and gastric cancer. Knockdown of UCA1 increases the sensitivity to tamoxifen in breast cancer through inhibition of Wnt/β-catenin pathway [154], and also promotes chemotherapy sensitivity to adriamycin in gastric cancer and accelerates cellular apoptosis pathway [155]. These findings of drug sensitivity in other cancers may provide promising therapeutic strategies for OSCC patients.

#### FOXCUT

FOXC1 upstream transcript (FOXCUT), a novel lncRNA associated with tumorigenesis, is transcribed from the upstream region of FOXC1 promoter. Studies have demonstrated that FOXC1 is a cancer-associated gene and its overexpression contributes to poor survival in the patients with breast cancer, OSCC, hepatocellular carcinoma, and so on [156–158]. Recently, the "lncRNA-mRNA pair" formed by lncRNA FOXCUT and mRNA FOXC1, has been verified to play a significant role in tumor progression. FOXC1 expression is positively correlated with FOXCUT’s in OSCC, and both of them are over-expressed in OSCC patients [157]. Knockdown of either FOXC1 or FOXCUT can remarkably inhibit cell proliferation, migration and invasion, and also reduce the expression levels of matrix metalloproteinases (MMPs) and VEGF-A [157].

#### FTH1P3

Ferritin heavy chain 1 pseudogene 3 (FTH1P3), a member of the ferritin heavy chain (FHC) gene family, has been confirmed to be responsible for progression and metastasis of OSCC cells [159]. A recent study has revealed that FTH1P3 is over-expressed in OSCC, and FTH1P3 overexpression contributes to the poorer OS of OSCC patients [160]. Ectopically expressed FTH1P3 can promote proliferative capacity and colony-forming ability in OSCC. Mechanistically, FTH1P3 acts as a ceRNA to sponge miRNA-224-5p, and then the down-regulated miR-224-5p activates frizzled 5 expression at the post-transcription level via alleviating the inhibition of the Wnt/β-catenin signaling [160]. The same mechanism was also found in breast cancer [161]. To conclude, both FTH1P3 and frizzled 5 are up-regulated in OSCC cells, and overexpression of frizzled 5 also has an oncogenic effect on OSCC progression [160, 161].

#### TUG1

The lncRNA, taurine up-regulated gene 1 (TUG1), has been found up-regulated in OSCC tissues and cell lines. The expression level of TUG1 in OSCC tissues is higher than that in normal adjacent tissues. TUG1 knockdown inhibits cell growth, proliferation, colony-forming ability and invasion, as well as induces cell apoptosis via inhibiting the Wnt/β-catenin signaling pathway in OSCC. These effects could be reversed by the LiCl-mediated activation of Wnt/β-catenin signaling pathway [162]. The findings indicate that TUG1 may serve as a novel target for OSCC treatment, and further study should be performed to evaluate it.

#### Application

Although clinical treatment has made some progress, the OS rate of OSCC is still just about 50–60% [163, 164]. This is mainly due to the difficulty of early detection. During the initial diagnosis, over 50% of cases of show lymph node metastasis [165, 166]. LncRNAs may act as early predictors in OSCC and be combined with surgery therapy.

**Figure 5:**
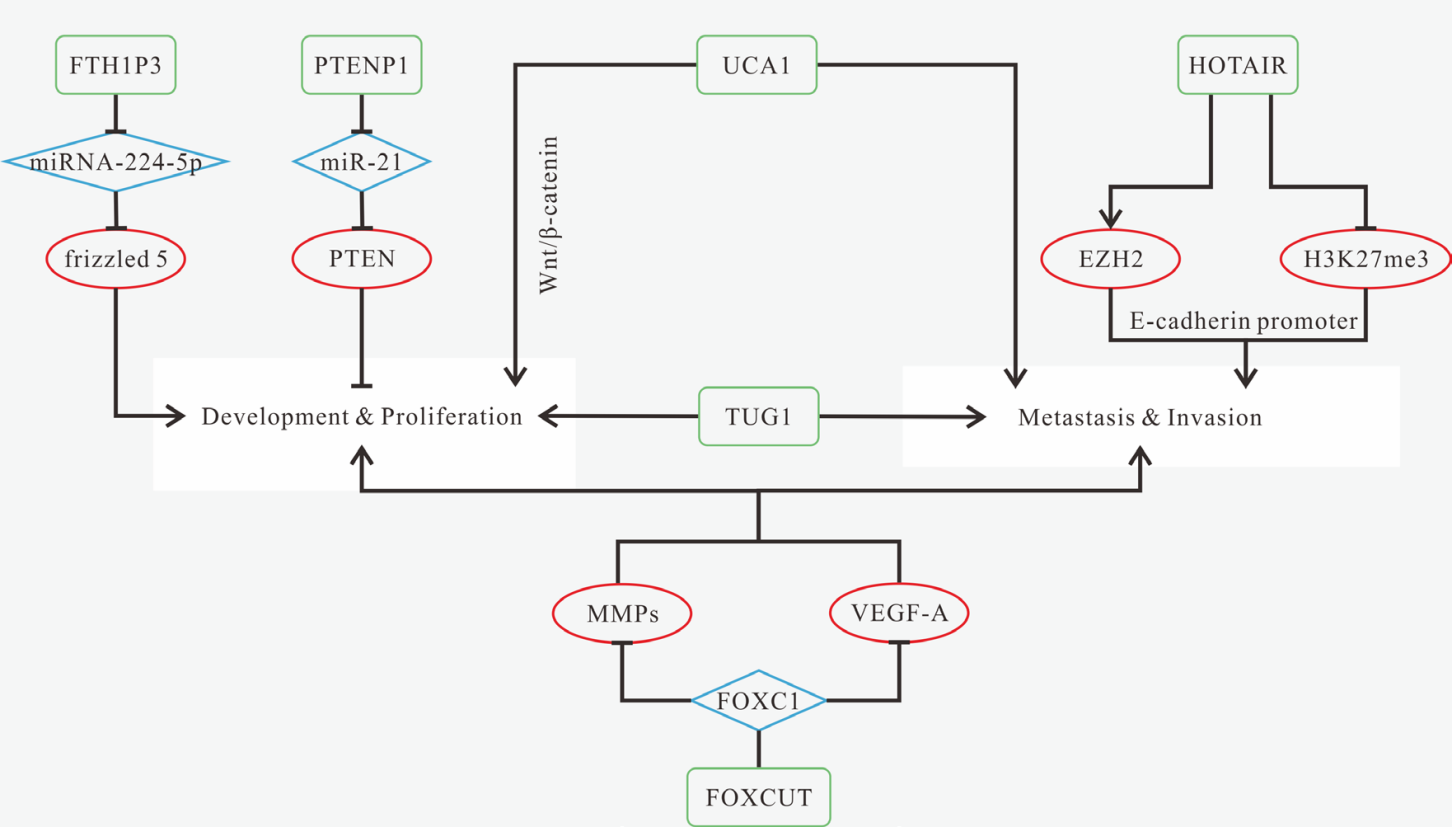
Mechanisms of lncRNAs in OSCC progression (**A**) FTH1P3 promotes proliferation and colony formation via acting as a miRNA sponge of miRNA-224-5p to activate frizzled 5 expression; (**B**) PTENP1 can inhibit proliferation and colony formation through acting as a ceRNA of miR-21 to increase PTEN expression; (**C**) up-regulated UCA1 promotes proliferation, metastasis and invasion, as well as inhibits cell apoptosis via modulating the Wnt/β-catenin signaling pathway; (**D**) HOTAIR promotes cell proliferation, migration and invasion, and inhibits apoptosis by recruiting EZH2; (**E**) TUG1 promotes cell growth, proliferation, colony-forming ability and invasion; (**F**) FOXCUT promotes cell proliferation, migration and invasion via increasing the expression levels of MMPs and VEGF-A, which is regulated by "FOXC1- FOXCUT pair".

Either up-regulation of CCAT2, FOXCUT, FTH1P3, HOTAIR, TUG1 and UCA1, or down-regulation of PTENP1 could be biomarkers for OSCC diagnosis [148, 150, 152, 157, 160, 162, 167]. The known mechanisms of OSCC-related lncRNAs are shown in Figure 5. HOTAIR expression level is associated with clinical stage [148, 167]. Although several lncRNAs alter their expression levels in cancer cells, only HOTAIR and MALALT-1 detected in the saliva has statistically difference and may act as a rapid and noninvasive diagnostic strategies [168]. Changes of the expression of downstream molecule miR-375 was also found in saliva [169]. MiR-375 is regulated by HNGA1 in HNSCC [167], and whether the same regulation exists in OSCC remains unclear.

Knockdown of CCAT2, FOXCUT, HOTAIR, TUG1 and UCA1 can suppress various aspects of tumor progression [148, 152, 157, 162]. Therapies targeted at FTH1P3-miR-224-5p-frizzled 5 axis or PTENP1-miR-21 axis might also be new treatment methods [160]. Patients with high expression of CCAT2, FOXCUT, FTH1P3 and HOTAIR or low expression of PTENP1 have poorer OS [148, 150, 157, 160]. CCAT2 is associated with tumor grade and distant metastasis. HOTAIR is related to tumor size [148, 167].

### LSCC-associated lncRNAs

#### NEAT1

Emerging evidence has presented that NEAT1 is over-expressed in HNC and participates in tumorigenesis and tumor progression through interactions with miRNAs. NEAT1 knockdown represses cell proliferation and invasion, induce G1 phase arrest and apoptosis in LSCC, as well as inhibit LSCC xenograft growth [170]. NEAT1 promotes LSCC progression because it could stimulate the expression of miR-107-targeting cyclin-dependent kinase 6 (CDK6) through the inhibition of miR-107 in LSCC [170]. On the contrary, overexpression of miR-107 significantly reduces CDK6 expression [170], which might be a potential therapeutic strategy for LSCC patients. The same molecular mechanism has been found in glioma stem cells [171]. Knockdown of NEAT1 can suppress glioma stem-like properties via modulating the miR-107/CDK6 pathway, but whether NEAT1 knockdown has the same effect on LSCC needs further investigation.

#### PVT1&LOC157273

Specific mRNAs are responsible for over-represented cell cycle and proteasome pathways in lymph node metastatic LSCC. PVT1 could decrease the expression of hsa-miR-1207-5p, and increase the expression of Glucose-6-phosphate dehydrogenase (G6PD) in LSCC cells, a downstream target of miR-1207-5p involved in the proteasome pathway [172]. A novel lncRNA LOC157273 expression has been reported increased in lymph node metastatic LSCC cell lines. It has been found to be correlated with the aberrant expression of hsa-miR-145-5p (down-regulated), as well as miR-145-5p’s downstream molecules CDK4 and SMC1A (up-regulated) [172]. The networks integrated by lncRNA PVT1, miR-1207-5p and mRNA G6PD, or composed of lncRNA LOC157273, miR-145-5p, as well as mRNAs CDK4 and SMC1A, play an important role in cell-cycle regulation, and might function as promising biomarkers for lymph node metastatic LSCC diagnosis.

#### HOTAIR

Phosphorylation of PRC2 complex member EZH2 at the post-translational level results in a stronger binding affinity between HOTAIR and EZH2 [173]. The expression level of HOTAIR is up-regulated in LSCC tissues [174, 175]. HOTAIR silencing impairs invasive ability, and induces apoptosis of LSCC cell line Hep-2 [174], but enhances proliferative capacity and attenuates the resistance to cis-platinum in AMC-HN8 cells [175]. HOTAIR-induced hypermethylation of CpG islands in PTEN promoter, a tumor suppressor, leads to a sharp decrease of PTEN expression in Hep-2 cells, indicating that HOTAIR might play an oncogenic role in LSCC progression through activation of the phosphatidylinositol 3-kinase (PI3K) signaling pathway induced by PTEN methylation [174, 176].

#### H19

Overexpression of H19 is associated with tumor grade, differentiation, neck lymph node metastasis, clinical stage, and OS [177]. As a miRNA sponge, lncRNA H19 involves in LSCC progression via modulating miR-148a-3p and DNA methyltransferase enzyme DNMT1 [177]. H19-induced decrease of miR-148a-3p eliminates the inhibitory effect of miR-148a-3p on DNMT1, thus promoting proliferation, metastasis and invasion via increasing DNA methylation in LSCC [177]. The activity of miR-148a-3p is required during the process above. However, miR148a-3p overexpression had little effect on the expression level of H19 [177].

#### Application

LSCC ranks the second among malignant lesion of upper aerodigestive tract. Treatment varies from surgery to a combination of surgery and radiotherapy [178]. However, the optimal treatment of LSCC remains unclear [179].

Knockdown of LINC00673, MALAT1 and UCA1 or overexpression of NKILA can inhibit cell migration [75, 136, 141, 142]. Overexpressed MEG3 can arrest cell cycle, inhibit tumor cell proliferation, and promote apoptosis [133]. AC007392.4 influences tumor cell growth and cell apoptosis rate [180]. As NKILA is associated with NF-κB, NF-κB inhibitor (Bay-117082 or JSH-23) can inhibit tumor cell metastasis [136].

**Figure 6:**
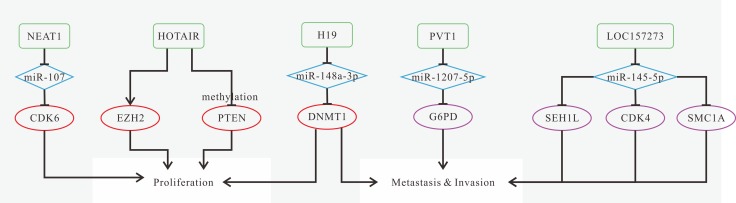
Mechanisms of lncRNAs in LSCC progression (**A**) NEAT1 promotes proliferation and invasion, and inhibits apoptosis via regulating miR-107/CDK6 axis; (**B**) HOTAIR promotes invasion and inhibits apoptosis by inducing PTEN methylation; (**C**) H19 promotes proliferation, migration and invasion by the lncRNA H19/miR-148a-3p/DNMT1 axis; (**D**) The network composed of lncRNA PVT1, miR-1207-5p, and mRNA G6PD might function as promising biomarkers for lymph node metastatic LSCC diagnosis; (**E**) LOC157273 plays an important role in cell-cycle regulation via the network integrated with miR-145-5p , and mRNAs CDK4 and SMC1A in lymph node metastatic LSCC.

AC007392.4, MEG3, NKILA are down-regulated in LSCC, while up-expression of HOTTIP, LINC00152, LINC00673, MALAT1, UCA1 is observed [75, 133, 136, 141, 142, 145, 146, 180]. Moreover, Highly expressed LINC00673 is associated with higher TNM stage [142]. MALAT1 and UCA1 are highly expressed in patients with LNM stage [75, 141]. For prognosis, patients with high-expression of HOTTIP, LINC00152 and LINC00673, or low expression of MEG3 and/or NKILA have poorer OS [133, 136, 142, 145, 146]. Highly expressed LINC00673 is associated with tumor size and relapse [142]. Down-regulated NKILA or highly expressed MALAT1 and UCA1 are associated with tumor metastasis, especially lymph node metastasis [75, 136, 141]. The known mechanisms of LSCC-related lncRNAs are shown in Figure 6.

## CONCLUSIONS

LncRNAs can modulate HNC tumorigenesis and development at diverse levels. They can participate in HNC progression via modulating gene expression at epigenetic, transcriptional, post-transcriptional and translational levels. For instance, lncRNA modulates tumor progression by altering the expression of proteins associated with cell proliferation, metastasis, and cell cycle. The regulatory role of lncRNA in various signaling pathways cannot be ignored. Additionally, lncRNAs can regulate miRNA-mediated downstream effector molecules by acting as a ceRNA for miRNAs, and the interactions between lncRNAs and miRNAs suggest a potential target for HNC treatment. Recently, the network composed of lncRNAs, mRNAs and miRNAs might provide an innovative idea for future research. Also, many of the mechanisms that have matured in other tumors have not yet been validated in HNC, and have led to new directions for future research. Radiotherapy and chemotherapy are still the most important treatment measures, but the effects of these methods still need to be improved, and patients’ discomfort caused by them is difficult to remove. Experiments have shown that some lncRNAs can improve the sensitivity of tumor cells to radiotherapy and chemotherapy.

In this review, we enumerate the functions and molecular mechanisms of dysregulated lncRNAs that have been confirmed to be involved in HNC until now. They may function as oncogenes or tumor inhibitors in HNC progression. Statistics and experiments have proved that some lncRNAs can serve as diagnostic, prognostic biomarkers, as well as provide promising therapeutic strategies for HNC patients. Since many lncRNAs have remained to be investigated, further research is required to determine precise, detailed functions and mechanisms of these lncRNAs in clinical practice.

## SUPPLEMENTARY MATERIALS TABLE





## Abbreviations

HNC: head and neck cancer
HNSCC: head and neck squamous cell carcinoma
HPV: human papillomavirus
NcRNAs: non-coding RNAs
LncRNAs: long non-coding RNAs
OSCC: oral squamous cell carcinoma
LSCC: laryngeal squamous cell carcinoma
TSCC: tongue squamous cell carcinoma
HSCC: Hypopharyngeal squamous cell carcinoma
NPC: nasopharyngeal carcinoma
TC: Thyroid cancer
PTC: papillary thyroid cancer
FTC: follicular thyroid cancer
ATC: anaplastic thyroid cancer
MTC: medullary thyroid cancer
DTC: differentiated thyroid cancer
PDTC: poorly differentiated thyroid cancer
HOTAIR: HOX transcript antisense RNA
RBPs: RNA binding proteins
ceRNA: competing endogenous RNA
IGF2: Insulin-like growth factor 2
LOI: loss of imprinting
JNA: juvenile nasopharyngeal angiofibroma
OS: overall survival
HNGA1: HNSCC glycolysis-associated 1
DFS: disease-free survival
VEGFA: vascular endothelial growth factor-A
GRP78: glucose-regulated protein 78
EMT: Epithelial-Mesenchymal Transition
EZH2: enhancer of zeste homolog 2
MALAT1: Metastasis-associated lung adenocarcinoma transcript 1
CSC: cancer stem cell
ANRIL: antisense non-coding RNA in the INK4 locus
CBX7: Chromobox 7
SP cells: side population cells
AFAP1-AS1: Actin filament associated protein 1 antisense RNA1
PARP: poly (ADP-ribose) polymerase
BANCR: BRAF-activated non-coding RNA
TSHR: thyroid-stimulating hormone receptor
PTCSC: papillary thyroid cancer susceptibility candidate
SNP: single nucleotide polymorphism
MYH9: Myosin-9
NEAT1: Nuclear Enriched Abundant Transcript 1
FAL1: Focally amplified lncRNA on chromosome 1
CDKN1A/p21: cyclin-dependent kinase inhibitor 1A
BMI1: BMI1 proto-oncogene
CDK: cyclin-dependent kinase
RB: retinoblastoma protein
GAS8-AS1: growth arrest-specific 8-antisense RNA 1
NAMA: noncoding RNA associated with mitogen-activated protein kinase pathway and growth arrest
IQGAP1: IQ motif containing GTPase activating protein 1
JAG1: jagged1
DKK1: Dickkopf-related protein 1
SPRR: small proline-rich proteins
MEG3: Maternally Expressed Gene 3
DNMT3B: DNA methyltransferase 3B
NKILA: Nuclear Factor-κB Interacting LncRNA
TNM: tumor node metastasis
IKK: IκB kinase
UCA1: Urothelial cancer-associated 1
LNM: lymph node metastasis
PRC2: polycomb repressive complex 2
LSD1: lysine-specific demethylase 1
CoREST: REST corepressor 1
REST: RE1-silencing transcription factor
H3K27: histone H3 lysine 27
H3K4: histone H3 lysine 4
FOXCUT: FOXC1 upstream transcript
MMPs: matrix metalloproteinases
FTH1P3: Ferritin heavy chain 1 pseudogene 3
FHC: ferritin heavy chain
TUG1: taurine up-regulated gene 1
CDK6: cyclin-dependent kinase 6
G6PD: Glucose-6-phosphate dehydrogenase
PI3K: phosphatidylinositol 3-kinase
