# Supplementary material for: Current research on head and neck cancer-associated long noncoding RNAs
Source: Oncotarget. 2017 Nov 22;9(1):1403–25. doi: 10.18632/oncotarget.22608 (PMC5787447; doi:10.18632/oncotarget.22608)
Supplement: Supplementary file 1 [file oncotarget-09-1403-s001.pdf]

Current research on head and neck cancer-associated long noncoding RNAs

SUPPLEMENTARY MATERIALS

Supplementary Table 1: Dysregulation and clinical implications of lncRNAs in HNC. See Supplementary_Table_1
