# Supplementary material for: Current research on head and neck cancer-associated long noncoding RNAs
Source: Oncotarget. 2017 Nov 22;9(1):1403–25. doi: 10.18632/oncotarget.22608 (PMC5787447; doi:10.18632/oncotarget.22608)
Supplement: Supplementary file 2 [file oncotarget-09-1403-s002.docx]

**Supplementary Table 1: Dysregulation and clinical implications of lncRNAs in HNC**

| **Cancer** | **LncRNA** | **Expression** | **Functions and potential mechanisms** | **Potential clinical implications** |
| --- | --- | --- | --- | --- |
| HNSCC | H19 | Up | Promotes cell viability, migration and invasion by miR-675 (the mature product of H19) overexpression in HNSCC [51]. | Knockdown of H19/miR-675 can inhibit tumor cell viability, migration and invasion [51].  Patients with high expression of H19 have lower overall survival and disease-free survival [51]. Highly expressed H19 is associated with higher relapse [51, 63]. |
|  | HNGA1 | Up | Promotes cell proliferation and glycolysis by acting as a ceRNA of miR-375 to increase SCL2A1 levels in HNSCC [52]. | Knockdown of HNGA1 can inhibit tumor cell proliferation [52]. Targeting HNGA1/miR-375/SCL2A1 axis could be a new therapeutic strategy [52].  HNGA1 is correlated with post-operative survival [52]. |
|  | HOTAIR | Up | Promotes metastasis by forming a feed-forward regulatory loop with HuR in HNSCC [43]. | Knockdown of HOTAIR can inhibit tumor cell viability, migration and invasion, as well as promote apoptosis [42, 43]. HOTAIR depletion can induce mitochondrial calcium uptake 1-dependent cell death [42].  Highly expressed HOTAIR is correlated with tumor size and lymph node metastasis [43]. Patients with high expression of HOTAIR have shorter overall survival [43]. |
|  | GAS5 | Unknown | Unknown mechanism [181]. | / |
|  | PANCR | Unknown | PANCR hypermethylation is related to an increased risk of death [61]. | The methylation status of PITX2 gene and its adjacent lncRNA PANCR might be effective predictors for overall survival [61]. |
|  | PTENP1 | Down | Inhibits growth, proliferation, colony formation, migration and invasion in HNSCC [55]. | Overexpressed PTENP1 can inhibit tumor cell proliferation, invasion and colony formation [55].  Patients with low expression of PTENP1 have poorer overall survival and disease-free survival [55]. |
| HSCC | AB209630 | Down | Inhibits growth, migration and invasion, and induces apoptosis in hypopharyngeal squamous cell carcinoma (HSCC) [182]. | Overexpressed AB209630 inhibits tumor growth, metastasis and invasion, and induces tumor apoptosis [182].  Patients with low expression of AB209630 have better overall survival and lower risk of death [182]. |
| LSCC | AC026166.2-001 | Down | Unknown mechanism [183]. | The area under ROC curve for AC026166.2-001 is 0.65, and the cut-off point of DCt is 11.23 [183].  Patients with low expression of AC026166.2-001 have lower overall survival [183]. |
|  | H19 | Up | Promotes proliferation, migration and invasion by the lncRNA H19/miR-148a-3p/DNMT1 axis in LSCC [184]. | Knockdown of H19 can inhibit tumor cell proliferation, migration and invasion [184]. Targeting H19/miR-148a-3p/DNMT1 axis could be a new therapeutic strategy [184].  Patients with high expression of H19 have lower overall survival [184]. |
|  | HOTAIR | Up | Promotes invasion and inhibits apoptosis by inducing PTEN methylation in LSCC [174]. | Highly expressed HOTAIR is correlated with clinical stage [185]. The combination of the serum expression of the HOTAIR and miR-21 is a valuable predicting biomarker, and the area under the ROC curve of combined examination of exosomal HOTAIR and miR-21 is 87.6% [185].  Knockdown of HOTAIR can inhibit tumor cell growth, invasion and promote apoptosis [174]. Knockdown of HOTAIR can increase the sensitivity of LSCC cells to cis-platinum [175].  Patients with high expression of HOTAIR have shorter overall survival [174]. Highly expressed HOTAIR is correlated with the risk of lymphatic metastasis [175]. |
|  | LOC157273 | Up | Plays an important role in cell-cycle regulation via the network integrated with miR-145-5p , and mRNAs CDK4 and SMC1A in lymph node metastatic LSCC [172]. | Highly expressed LOC157273 is correlated with lymph node metastasis [172]. |
|  | MALAT1 | Up | Promotes cell proliferation and inhibits apoptosis in LSCC [77]. | Knockdown of MALAT-1 can suppress tumour growth and induce apoptosis [77]. |
|  | NEAT1 | Up | NEAT1 knockdown inhibits proliferation and invasion, and induces G1 phase arrest and apoptosis via regulating miR-107/CDK6 axis in LSCC [170]. | Highly expressed NEAT1 is correlated with advanced clinical stage [170].  Knockdown of NEAT1 can inhibit tumor cell proliferation and induce cell cycle arrest and apoptosis [170].  Highly expressed NEAT1 is correlated with lymph node metastasis [170]. |
|  | PVT1 | Up | The network composed of lncRNA PVT1, miR-1207-5p, and mRNA G6PD might function as promising biomarkers for lymph node metastatic LSCC diagnosis [172]. | Knockdown of PVT1 can inhibit TSHR expression [108]. Targeting PVT1/miR-1207-5p/G6PD or PVT1/hsa-miR-145-5p/SMC1A&CDK4 axis could be new therapeutic strategies [172].  Highly expressed PVT1 is correlated with lymph node metastasis [108]. |
|  | RP11169D4.1-001 | Down | Unknown mechanism [183]. | The area under ROC curve for AC026166.2-001 is 0.67, and the cut-off point of DCt is 10.53 [183].  Down-regulated RP11169D4.1-001 is correlated with neck lymph node metastasis [183]. Patients with low expression of RP11169D4.1-001 have lower overall survival [183]. |
| NPC | AFAP1-AS1 | Up | Promotes metastasis by increasing expression of AFAP1 and some cytoskeleton-regulated proteins in NPC [89]. | Highly expressed AFAP1-AS is correlated with distant tumor metastasis [89].  Patients with high expression of AFAP1-AS1 have poorer overall survival and relapse-free survival [89]. |
|  | ANRIL | Up | Enhances the proliferative and transforming capacity, reprograms cell glucose metabolism and induces SP cells possibly through activation of the mTOR signal pathway in NPC [83]; be associated with tumorigenicity and cisplatin sensitivity by negatively regulating miR-let-7a in NPC [84]. | ANRIL is highly expressed in advanced-stage cancer [83].  Knockdown of ANRIL can inhibit tumorigenicity and enhance DDP-induced cytotoxicity by regulating miRNA let-7a [84].  Patients with high expression of ANRIL have longer overall survival and shorter disease-free survival [83]. |
|  | ENST00000438550 | Up | Unknown mechanism [186]. | Patients with low expression of ENST00000438550 have higher disease-free survival [186]. |
|  | H19 | Up | Inhibits E-cadherin expression, induces EMT, and promotes migration and invasion via modulating the miR-630/EZH2 axis in NPC [67]. | Knockdown of H19 can inhibit tumor cell invasion [67]. |
|  | HNF1A-AS | Up | Promotes cell proliferation and metastasis by accelerating the EMT in NPC [187]. | Knockdown of HNF1A-AS can inhibit tumor cell proliferation and metastasis [187]. |
|  | HOTAIR | Up | Promotes cell growth and angiogenesis by inducing VEGFA expression directly or indirectly in NPC [65]. | Knockdown of HOTAIR can inhibit tumor cell proliferation and promote apoptosis [64, 65]. Silencing of HOTAIR can inhibit tumorigenesis by anti-proliferation and anti-angiogenesis [65].  Patients with high expression of HOTAIR have poorer local recurrence-free survival, overall survival and disease-free survival [64]. |
|  | LET | Down | Represses proliferation and induces apoptosis; low LET expression was induced by EZH2-mediated H3K27 histone methylation in the LET promoter region in NPC [92]. | Elevated LET expression suppresses cell proliferation and induces apoptosis of NPC cells [92].  Down-regulated LET is correlated with tumor size and poorer prognosis [92]. |
|  | LINC0086 | Down | Inhibits proliferation and promotes apoptosis by increasing the expression of miR-214 in NPC [93]. | Down-regulated in tumor tissues and serum [93]. LINC0086 expression is associated with clinical stage [93].  Targeting LINC0086/miR-214 axis could be a new therapeutic strategy [93].  LINC0086 expression is associated with lymph node metastasis [93]. Patients with high expression of LINC0086 have higher survival rate [93]. |
|  | LINC00312 | Down | Unknown mechanism [96]. | Highly expressed LINC00312 is negatively correlated with tumor size and positively correlated with lymph node metastasis [96].  Patients with low expression of LINC00312 have lower disease-free survival and overall survival [96]. |
|  | LOC401317 | Unknown | Inhibits NPC cell cycle progression by up-regulating p21 and down-regulating cyclin D1 and E1; promotes apoptosis by activating PARP and caspase 3. LncRNA LOC401317 expression is directly regulated by p53 [94]. | Down-regulated LOC401317 can arrest cell cycle and promote apoptosis [94]. |
|  | MALAT1 | Up | Promotes cell proliferation, invasion, and metastasis in NPC [78]; promotes cancer stem cell activity and induces radioresistance to up-regulate slug by reducing miR-1 activity in NPC [72]; | Highly expressed MALAT1 is associated with clinical stage [72].  Knockdown of MALAT1 can increase radiation sensitivity *in vitro and in vivo* by regulating miR-1/slug axis [72]. Knockdown of MALAT1 can inhibit tumor cell proliferation, migration and invasion, as well as promote apoptosis [78].  Patients with high expression of MALAT1 have poorer overall survival [72]. |
|  | n375709 | Up | Inhibition of lncRNA n375709 increases the paclitaxel sensitivity [97]. | Downregulation of n375709 can increase the sensitivity of NPC cell lines (5-8F and 6-10B) to paclitaxel [97]. |
|  | ROR | Up | Decreases the apoptosis rate and promotes cell proliferation; promotes metastasis and invasion by inducing EMT in NPC; enhances the chemotherapy resistance ability of NPC cells by inhibiting the p53 pathways in NPC [86]. | Knockdown of ROR can inhibit tumor cell proliferation and metastasis, as well as promote apoptosis [86]. Downregulation of ROR can reduce chemotherapy resistance ability [86]. |
| OSCC | CCAT2 | Up | Promotes cell proliferation, migration, and invasion in OSCC [167]. | Knockdown of CCAT2 can repress tumor cell proliferation, migration and invasion [167].  CCAT2 is associated with tumor grade and distant metastasis [167]. Patients with high expression of CCAT2 have lower overall survival [167]. |
|  | FOXCUT | Up | Promotes cell proliferation, migration and invasion via increasing the expression levels of MMPs and VEGF-A, which is regulated by “FOXC1- FOXCUT pair” in OSCC [157]. | Knockdown of FOXCUT can inhibit tumor cell proliferation and migration [157].  Patients with high expression of FOXCUT have lower overall survival [157]. |
|  | FTH1P3 | Up | Promotes proliferation and colony formation via acting as a miRNA sponge of miRNA-224-5p to activate frizzled 5 expression in OSCC [160, 161]. | Targeting FTH1P3/miR-224-5p/fizzled 5 axis could be a new therapeutic strategy [160].  Patients with high expression of FTH1P3 have lower overall survival [160]. |
|  | HOTAIR | Up | Promotes cell growth, proliferation and colony formation, migration and invasion, and inhibits apoptosis by recruiting EZH2 in OSCC [148, 149]. | HOTAIR expression level is associated with clinical stage [148]. HOTAIR is highly expressed in saliva of patients with LNM stage [188].  Knockdown of HOTAIR can inhibit tumor cell growth, migration and invasion, as well as induce apoptosis [148, 149].  HOTAIR expression level is associated with tumor size [148]. Patients with high expression of HOTAIR have shorter overall survival and disease-free survival [149]. |
|  | MALAT1 | Up | Promotes tumor growth and metastasis by inducing EMT in OSCC [79]. | Patients with low MALAT1 expression have increased overall survival [79]. |
|  | PTENP1 | Down | PTENP1 can inhibit proliferation and colony formation through acting as a ceRNA of miR-21 to increase PTEN expression in OSCC [150]. PTENP1 overexpression induces cell cycle arrest at S-G2/M phase via restraining the AKT pathway in OSCC [150]. | Targeting PTENP1/miR-21 axis could regulate tumor cell proliferation and reduce tumorigenicity [150].  Patients with low expression of PTENP1 have poorer overall survival [150]. |
|  | TUG1 | Up | TUG1 knockdown inhibited cell growth, proliferation, colony-forming ability and invasion, as well as induced cell apoptosis [162]. | Knockdown of TUG1 can inhibit tumor cell proliferation, invasion and colony formation ability, as well as promote apoptosis [162]. |
|  | UCA1 | Up | UCA1 silencing represses proliferation, metastasis and invasion, as well as induces cell apoptosis via modulating the Wnt/β-catenin signaling pathway in OSCC [152]. | Knockdown of UCA1 can inhibit tumor cell proliferation, migration and invasion [152]. |
| TC  (Thyroid Cancer) | BANCR | Up/Down | BANCR is up-regulated in PTC-derived cell line IHH-4, while BANCR knockdown inhibits cell proliferation and induces apoptosis and cell cycle arrest at G0/G1 phase through downregulation of cyclin D1 and TSHR [98]. BANCR is down-regulated in PTC cell lines (TPC-1, K1, and BCPAP), while BANCR overexpression inhibits cell proliferation and invasion, and induces apoptosis through inactivation of ERK and p38 [100]. | Up-regulated in tumor tissues and PTC cell line IHH-4 [98, 99]; down-regulated in tumor tissues and PTC cell lines (TPC-1, K1, and BCPAP) [100].  Knockdown of BANCR can inhibit proliferation, activate autophagy and increase apoptosis of IHH-4 cells [98]. Knockdown of BANCR can inhibit TSHR [99]. Overexpressed BANCR can inhibit tumor cell proliferation and metastasis, as well as promote apoptosis in PTC cell lines (TPC-1, K1, and BCPAP) [100].  Down-regulated BANCR is associated with poor prognosis (tumor size, presence of multifocal lesions and advanced pathological stage) in PTC cell lines (TPC-1, K1, and BCPAP) [100]. |
|  | ENST00000426615 | Up | Promotes proliferation and cell motility in PTC [189]. | Knockdown of ENST00000426615 can inhibits proliferation and cell motility in PTC [189]. |
|  | ENST00000537266 | Up | Promotes proliferation in PTC [189]. | Knockdown of ENST00000537266 can inhibits proliferation in PTC [189]. |
|  | FAL1 | Up | Promotes tumor proliferation and generates aggressive tumor behavior such as multifocality through the increase of cyclin D1, E2F1, E2F2 and VEGF-A in PTC [117]. | Highly expressed FAL1 may be associated with PTC malignant behavior and aggressive features [117].  Highly expressed FAL1 increases the risk of multifocality [117]. |
|  | GAS8-AS1 | Down | Inhibits cell viability in PTC [118]. | Patients with GAS8-AS1 mutations are associated with advanced PTC disease [118]. |
|  | HOTAIR | Up | HOTAIR SNP rs920778 genetic variants are significantly associated with PTC initiation via engaging in the regulation of HOTAIR expression [52]. | HOTAIR depletion can inhibit tumor cell proliferation [122]. |
|  | LOC100507661 | Up | Promotes the proliferation, migration and invasion in TC [127]. | Knockdown of LOC100507661 can inhibit tumor cell proliferation, migration and invasion [127]. |
|  | MALAT1 | Up/Down | MALAT1 is highly expressed in both non-cancerous thyroid tissues and PTC tissues, whereas it is down-regulated in ATCs and PDTCs [71]. Promotes proliferation and invasion by up-regulating the expression of IQGAP1 in FTC [76]. | Targeting MALAT1/ IQGAP1 axis could be a new therapeutic strategy [76]. |
|  | NAMA | Down | Decreased BRAF expression, inactivated MAP pathway or DNA damage can result in the induction of NAMA in PTC [119]. Upregulation of NAMA induces cell growth arrest and apoptosis via modulating Raf– MEK–ERK signaling pathway in PTC [119]. | Overexpressed NAMA can induce cell cycle arrest and apoptosis [119]. |
|  | NEAT1 | Up | Promotes migration and invasion, as well as increases the expression of β-catenin through the reduction of miR-214 in PTC [126]. | Knockdown of NEAT1 can inhibit tumor cell survival, migration and invasion [126]. |
|  | NONHSAT037832 | Down | Unknown mechanism [128]. | Down-regulated NONHSAT037832 is associated with tumor size and lymph node metastasis [128]. |
|  | NONHSAT076754 | Up | Promotes cell invasion and lymph node metastasis in PTC [125]. | Knockdown of NONHSAT076754 can inhibit cell invasion and lymph node metastasis [125].  Highly expressed NONHSAT076754 is associated with lymph node metastasis [125]. |
|  | PTCSC2 | Down | Influences the expression of some genes involved in cancer and the cell cycle in PTC [103]. The risk allele [A] of SNP rs965513 is significantly correlated with the low expression levels of PTCSC2*,* FOXE1 and TSHR in non-tumor thyroid tissues, thus resulting in high PTC risk. | / |
|  | PTCSC3 | Down | Influences gene expression, DNA replication, recombination and repair, as well as tumor cell growth, motility, death and tumor morphology in PTC [104]; inhibits cell growth, promotes apoptosis and arrests cell cycle at G1/S and G2/M phases in thyroid cancer [106]. | / |
|  | PVT1 | Up | Silencing of PVT1 represses proliferation, induces G0/G1 cell cycle arrest and decreases cyclin D1 expression through reducing recruitment of EZH2 and modulating TSHR in TC [108]. | / |
| TSCC | AC007392.4 | Down | Promotes proliferation and inhibits apoptosis in TSCC [180]. | Influences tumor cell growth and cell apoptosis rate [180]. |
|  | HOTTIP | Up | Unknown mechanism [146]. | HOTTIP is associated with clinical stage, T stage and distant metastasis status [146].  Patients with high expression of HOTTIP have lower overall survival [146]. |
|  | LINC00152 | Up | Unknown mechanism [145]. | Patients with high expression of LINC00152 have poorer overall survival [145]. |
|  | LINC00673 | Up | Promotes migration and invasive properties in TSCC [142]. | Highly expressed LINC00673 is associated with higher TNM stage [142].  Knockdown of LINC00673 can inhibit cell migration and invasion [142].  Highly expressed LINC00673 is associated with tumor size and relapse [142]. Patients with high expression of LINC00673 have shorter overall survival and relapse-free survival [142]. |
|  | Lnc-AL355149.1-1 | Down | Unknown mechanism [190]. | / |
|  | Lnc-MBL2-4:3 | Up | Unknown mechanism [190]. | / |
|  | Lnc-PPP2 R4-5 | Up | Unknown mechanism [190]. | / |
|  | Lnc-SPRR2D-1 | Up | Unknown mechanism [190]. | / |
|  | MALAT1 | Up | MALAT1 overexpression promotes tumor growth and invasion by increasing JAG1 expression resulted from the reduction of miR-124 in TSCC [73]; modulates tumor migratory potential partially through the regulation of SPRR in TSCC [75]; induces EMT and inhibits apoptosis via modulating Wnt/β-catenin signaling pathway in TSCC [74]. | MALAT1 is highly expressed in patients with LNM stage [75].  Knockdown of MALAT1 can influence tumor cell metastasis [75].  Highly expressed MALAT1 is associated with lymph node metastasis [75]. |
|  | MEG3 | Down | MiR-26a-mediated reduction of DNMT3B expression results in MEG3 overexpression, and inhibits cell proliferation, prevents cell cycle and promotes apoptosis in TSCC [133]. | Overexpressed MEG3 can arrest cell cycle, inhibit tumor cell proliferation, and promote apoptosis [133].  Patients with low expression of MEG3 have poorer overall survival [133]. |
|  | NKILA | Down | NKILA represses tumor cell migration and invasion via inhibiting IκBα phosphorylation induced by IKK, NF-κB activation and the induction of EMT process in TSCC [136]. | Down-regulated NKILA is associated with advanced clinical stage [136].  Overexpressed NKILA can inhibit tumor cell migration and invasion [136]. NF-κB inhibitor (Bay-117082 or JSH-23) can inhibit tumor cell migration capacity [136].  Down-regulated NKILA is associated with lymph node metastasis [136]. Patients with low expression of NKILA have poorer overall survival [136]. |
|  | UCA1 | Up | Promotes migration in TSCC [141]. | UCA1 is highly expressed in patients with LNM stage [141].  Knockdown of UCA1 can inhibit cell invasion [141].  Highly expressed UCA1 is associated with lymph node metastasis [141]. |
